# Block of Voltage-Gated Sodium Channels as a Potential Novel Anti-cancer Mechanism of TIC10

**DOI:** 10.3389/fphar.2021.737637

**Published:** 2021-10-21

**Authors:** Eva Fuchs, David Alexander Christian Messerer, Georg Karpel-Massler, Michael Fauler, Thomas Zimmer, Bettina Jungwirth, Karl Josef Föhr

**Affiliations:** ^1^ Department of Anesthesiology and Intensive Care Medicine, University Hospital of Ulm, Ulm, Germany; ^2^ Department of Neurological Surgery, University Hospital of Ulm, Ulm, Germany; ^3^ Institute of General Physiology, University of Ulm, Ulm, Germany; ^4^ Institute of Physiology, University Hospital of Jena, Jena, Germany

**Keywords:** TIC10, ONC201, voltage-gated sodium channel, drug repurposing, tumor therapy

## Abstract

**Background:** Tumor therapeutics are aimed to affect tumor cells selectively while sparing healthy ones. For this purpose, a huge variety of different drugs are in use. Recently, also blockers of voltage-gated sodium channels (VGSCs) have been recognized to possess potentially beneficial effects in tumor therapy. As these channels are a frequent target of numerous drugs, we hypothesized that currently used tumor therapeutics might have the potential to block VGSCs in addition to their classical anti-cancer activity. In the present work, we have analyzed the imipridone TIC10, which belongs to a novel class of anti-cancer compounds, for its potency to interact with VGSCs.

**Methods:** Electrophysiological experiments were performed by means of the patch-clamp technique using heterologously expressed human heart muscle sodium channels (hNav1.5), which are among the most common subtypes of VGSCs occurring in tumor cells.

**Results:** TIC10 angular inhibited the hNa_v_1.5 channel in a state- but not use-dependent manner. The affinity for the resting state was weak with an extrapolated K_r_ of about 600 μM. TIC10 most probably did not interact with fast inactivation. In protocols for slow inactivation, a half-maximal inhibition occurred around 2 µM. This observation was confirmed by kinetic studies indicating that the interaction occurred with a slow time constant. Furthermore, TIC10 also interacted with the open channel with an affinity of approximately 4 µM. The binding site for local anesthetics or a closely related site is suggested as a possible target as the affinity for the well-characterized F1760K mutant was reduced more than 20-fold compared to wild type. Among the analyzed derivatives, ONC212 was similarly effective as TIC10 angular, while TIC10 linear more selectively interacted with the different states.

**Conclusion:** The inhibition of VGSCs at low micromolar concentrations might add to the anti-tumor properties of TIC10.

## Introduction

TIC10, also known as ONC201 or NSC350625, belongs to a novel class of anti-cancer compounds called imipridones (Wagner et al., 2017). It operates independently from p53 and provides anti-proliferative and pro-apoptotic effects against a broad range of tumors, including hematological malignancies, glioblastoma, colorectal cancer or cancer stem cells (Allen et al., 2016). Altogether, a favorable side effect profile was observed in clinical trials (Stein et al., 2019; Arrillaga-Romany et al., 2020). Meanwhile, several derivatives of TIC10 were generated with in part significantly higher potency regarding inhibition of cell growth of different types of human cancer cells. Nevertheless, TIC10 still represents the most advanced imipridone with respect to the pre-clinical and clinical investigations (Ishida et al., 2018; Ma et al., 2019). So far, different modes of action were described for TIC10: The antineoplastic activity of TIC10 was first identified in a drug screen searching for compounds that induce TRAIL. As a consequence, the name of TIC10 is derived from this function as TRAIL-inducing compound 10 (Wagner et al., 2017). Recently, the mitochondrial caseinolytic protease P has been proposed and identified as the direct molecular target of TIC10 (Ishizawa et al., 2019; Wang and Dougan, 2019). In addition, using a newly developed algorithm, TIC10 has been predicted to exert a highly specific interaction with dopamine D_2_ receptors (Kline et al., 2016). Meanwhile, the D_2_ antagonizing property of TIC10 has been confirmed, which in turn has been demonstrated to have anti-tumor effects (Allen et al., 2016). As the imipridones were initially developed as anti-seizure medication, voltage-gated sodium channels (VGSCs) might also be potential targets (Kline et al., 2016; Pruss et al., 2020). Therefore, it is tempting to speculate that a link to a possible anti-tumor activity exists, as blockers of VGSCs are known to affect tumor metastasis (Djamgoz et al., 2019).

VGSCs are composed from one out of nine different pore forming *α*-subunits which are associated with no, one or two out of four auxiliary ß-subunits (Catterall et al., 2005; Patino and Isom, 2010). The *α*-subunits are designated as Na_v_1.1 to Na_v_1.9 according to their phylogeny. Other diversity arises from alternative splicing of mRNA and post-translational modifications. The distribution of the different subunits is tissue specific. Na_v_1.4 and Na_v_1.5 are predominantly found in skeletal and heart muscle cells, whereas Na_v_1.1 to Na_v_1.3 and Na_v_1.6 to Na_v_1.9 are found in neurons. According to pharmacological parameters the individual channels are classified as TTX-sensitive or TTX-resistant, depending on the concentration of tetrodotoxin (TTX) which is required for their blockage. Further classifications are based on different subunit-specific electrophysiological properties (Theile and Cummins, 2011).

During a screening of different anti-cancer agents, we identified TIC10/ONC201 as a potent blocker of VGSCs. In the present work, we investigated the potential of TIC10 to block VGSCs, which might add a supplementary effect on therapy. So far, almost all VGSC subtypes have been detected in different tumor entities (Roger et al., 2015). Most prevalent are the Na_v_1.7 and the Na_v_1.5 subtype. The latter was found to be present in a huge variety of diverse malignancies including breast cancer, colon cancer, ovarian cancer, melanoma, astrocytoma, or neuroblastoma (Djamgoz et al., 2019). Therefore, we performed the underlying experiments with the VGSC preferentially expressed in the heart muscle (hNa_v_1.5). This study reports about details regarding the mechanism of the underlying interaction.

## Methods

### Cell Culture

The tsA201 cell line is a transformed human kidney 293 (HEK293) cell line stably expressing an SV40 temperature-sensitive T antigen (Sigma-Aldrich no. 85120602). TsA201 cells were cultured at 37°C in a humidified atmosphere at 95% air and 5% CO_2_ in MEM (minimum essential medium) supplemented with 50 U/ml penicillin, 50 μg/ml streptomycin (Gibco, Eggenstein, Germany), 2 mM l-glutamine (Boehringer, Mannheim, Germany), and 10% fetal calf serum (Gibco). The cells were grown on polyornithine-coated culture dishes to 40% confluency and transfected using the TransFectin LipidReagent kit (Bio-Rad, München, Germany). The construction of the plasmid pTSV40G-hNa_v_1.5 encoding wild-type hNa_v_1.5 was described previously (Walzik et al., 2011). This plasmid allows for a simplified selection of transfected cells as EGFP is simultaneously produced from a separate expression cassette. Mutant channels (hNa_v_1.5_WCW and hNa_v_1.5_WCW_F1760K) were obtained by respectively modified oligonucleotides and overlapping PCR. The PCR fragments were inserted into the pTSV40G-hNa_v_1.5 background using restriction sites Age/BsaBI (for WCW) and BstEII/SpeI (for F1760K), resulting in pTSV40G-hNa_v_1.5_WCW and pTSV40G-hNa_v_1.5_WCW_F1760K.

### Electrophysiology

Electrophysiological experiments were performed as previously described (Ludolph et al., 2010; Föhr et al., 2021). Briefly, tsA201 cells were used for experiments 24–48 h after transfection. Membrane currents were recorded in the whole-cell recording mode using an EPC-9 amplifier and Patchmaster software (v2x73; HEKA, Lambrecht, Germany (Hamill et al., 1981)). Before recording, cells were rinsed twice with an extracellular standard solution containing (in mM): 140 NaCl, 5 KCl, 1.5 CaCl_2_, 1.0 MgCl_2_, 10 glucose and 12 HEPES (4-(2-hydroxyethyl)piperazine- 1-ethanesulfonic acid; pH 7.3). For establishing activation curves, the concentration of NaCl was reduced to 30 mM to achieve current amplitudes in the range of 1 nA. Lacking cations were replaced equimolar by N-Methyl-d-glucamine (NMDG). Patch pipettes were drawn from borosilicate glass with tip resistances of about 2 MΩ when filled with (in mM): 125 CsF, 10 NaCl, 10 EGTA (ethylene glycol-bis(2-aminoethylether)-*N,N,N′,N′*-tetraacetic acid), 10 HEPES; pH 7.2. To improve sealing, tips were briefly dipped into 2% dimethylsilane dissolved in dichloromethane.

Unless otherwise stated, the membrane potential was held at −140 mV from which channel activations were elicited by brief depolarizing pulses to −20 mV of 5 ms duration. In order to minimize voltage errors, the series resistance was compensated up to 80%. Cells with currents larger than 6 nA were excluded from evaluation. Specific protocols are illustrated in figure legends where appropriate.

### Drug Application

The medium in the dish (1.5 ml) was continuously exchanged using a “global” bath perfusion with the inflow set to 4.5 ml/min and the outflow removing any excess fluid. Reagents were applied locally to the cells by the L/M-SPS-8 superfusion system (List, Darmstadt, Germany). Switching between the eight channels of the superfusion system was controlled by magnetic valves. The local inlet (tip of an eight-barreled pipette) was positioned at a distance of 50–100 μm upstream and the local outlet at about 300 μm downstream of the patch pipette. A constant flow rate of control and test solutions (1 ml/min) was achieved by means of a pressure control system (MPCU-3, Lorenz, Göttingen, Germany). The time of solution exchange was estimated from the changes in the liquid junction potential to be about 1 ms. If not otherwise stated, drugs were pre-applied for 20 s before starting the experiments.

### Chemicals

**FIGURE 1:**
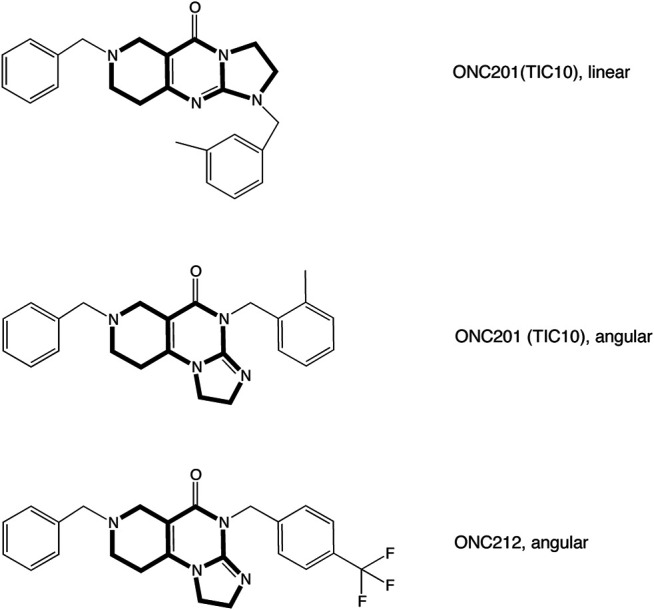
Structural formulae of analyzed imipridones. The graph illustrates the structural formula of TIC10 also known as ONC201 and ONC212. TIC10 linear and TIC10 angular are isomeres which differ in the arrangement of the tricyclic structure.

Trypsin was obtained from Biochrom AG, Berlin, Germany. DNAse 1 was obtained from Invitrogen, Carlsbad, Germany; fetal calf serum was obtained from HyClone, Perbio Science, Bonn, Germany. Poly-l-ornithine was purchased from Sigma-Aldrich, Schnelldorf, Germany. TIC10 angular was from Sigma-Aldrich Chemie GmbH, Steinheim, Germany, TIC10 linear from AOBIUS, United Stated and ONC212 from Selleckchem, United Stated (Figure 1); 10 mM stocks of these drugs were prepared with DMSO. All other chemicals were obtained from Sigma-Aldrich Chemie GmbH, Steinheim, Germany.

### Data Analysis and Statistics

#### Concentration-Inhibition Curves

Concentration-inhibition curves for estimating K_r_ (*IC*_*50*_ at −140 mV; Figure 3), K_i_ (Figures 8, 13, 14), K_o_ (Figures 12, 17) or *K*_*app*_ (Figure 9) were fitted to the Hill-equation.

$$\frac{I_{D}}{I_{C}}=\frac{1}{1+{\left(\frac{\left[D\right]}{IC_{50}}\right)}^{n}}$$ (1)

*I*_*D*_ and *I*_*C*_ are the current amplitudes in the presence and absence of the drug, where [*D*] is the concentration of the drug. *IC*_*50*_ represents the concentration of the blocker that causes 50% inhibition and *n* is the Hill-coefficient.

#### Voltage-dependent Behavior

Voltage-dependence of activation was calculated in two steps: First, changes in driving force owing to the different test potentials were considered by calculating the conductance *g* according to

$$g=\frac{1}{V-E_{Na}}$$ (2)

Thereafter, normalized data were fitted with a Boltzmann equation of the form:

$$\frac{g}{g_{\max}}=\frac{1}{1+e\left(\frac{V_{50}-V}{k}\right)}$$ (3)

Voltage-dependence of fast inactivation were fitted using a Boltzmann equation of the form:

$$\frac{I}{I_{\max}}=\frac{1}{1+e\left(\frac{V-V_{50}}{k}\right)}$$ (4)

In case of slow inactivation, Eq. 4 was extended by the additional parameter (*S*) which considers the steady-state level of incomplete inactivation.

$$\frac{I}{I_{\max}}=\left(1-S\right)\left(\frac{1}{1+e\left(\frac{V-V_{50}}{k}\right)}\right)+S$$ (5)

Abbreviations used for voltage-dependent parameters: *V* and *V*_*50*_ are the actual clamp potential and the potential at which half-maximal current *(I)* or conductance *(g)* occurs. *E*_*Na*_ indicates the reversal potential for sodium ions, which was experimentally determined for each cell. The slope factor is given by *k*.

#### Interaction With the Inactivated State

The interaction with the inactivated state cannot be directly analyzed, as this state is non-conducting. Therefore, different indirect approaches have been developed for this purpose.

1) In the first set of experiments, the drug-induced shift (*ΔV*_*50*_) of the inactivation curves on the potential axis was used to estimate the affinity to the inactivated state *K*_*i*_. For this purpose, the relative shift of the inactivation midpoints was plotted versus the concentration of TIC10. Data were fitted with the following equation (Bean et al., 1983):

$$\Delta V_{50}=k*ln\left(\frac{1+\frac{\left[D\right]}{K_{r}}}{1+\frac{\left[D\right]}{K_{i}}}\right)$$ (6)

*K*_*r*_ and *K*_*i*_ denote the affinity for the resting and inactivated state, respectively. Other identifiers have the same meaning as before.

2) Estimation of apparent binding constants:

The affinity to the inactivated state (*K*_*i*_) was calculated here according to (Bean et al., 1983) as:

$$\frac{1}{K_{app}}=\frac{h}{K_{r}}+\frac{\left(1-h\right)}{K_{i}}$$ (7)

*K*_*app*_ is the apparent affinity determined at a selected membrane potential at which the amount of non-inactivated channels (resting channels) is given by *h* (determined from the preceding inactivation curve). *K*_*r*_ is the affinity for the resting state, here 600 µM.

3) Time and concentration-dependent development of block:

The time constants (*τ*_*i*_) of block development for different concentrations of TIC10 were estimated by double exponential fits of the form:

$$\frac{I}{I_{\max}}=S+a_{1}*e^{\left(\frac{-t}{\tau_{1}}\right)}+a_{2}*e^{\left(\frac{-t}{\tau_{2}}\right)}$$ (8)

*t* denotes the time of inactivation, *τ*_*i*_ are individual time constants and *a*_*i*_ represents the relative contribution of the two terms, with *S + a*_*1*_ *+ a*_*2*_ *= 1*.

Association and dissociation rates:

Association (*k*_*on*_ in µM^−1^s^−1^) and dissociation rates (*k*_*off*_ in s^−1^) were estimated from the slope and the *y*-intercept of a linear regression where the inverse of the fast time constants (*1/τ*) was plotted against the drug concentration [*D*] (Kuo et al., 1997).

$$\frac{1}{\tau }=k_{off}+k_{on}*\left[D\right]$$ (9)

The corresponding dissociation constants were calculated according to:

$$K_{d}=\frac{k_{off}}{k_{on}}$$ (10)

#### Recovery From Inactivation

Current amplitudes were normalized to the maximum amplitude of *I*_*Na*_ in the absence and presence of TIC10. Recovery time constants were estimated from double or triple exponential fits according to:

$$\frac{I}{I_{\max}}=1-a_{1}*e^{\left(\frac{-t}{\tau_{1}}\right)}-a_{2}*e^{\left(\frac{-t}{\tau_{2}}\right)}-a_{3}*e^{\left(\frac{-t}{\tau_{3}}\right)}$$ (11)

Variable identifiers have the same meaning as before, with *a*_*1*_ *+ a*_*2*_ *+ a*_*3*_ *= 1*.

All curve-fitting procedures were performed using SigmaPlot 13.0 (Sysstat, San Jose, California, United Stated).

#### Nomenclature

Upon activation of Nav1.5 channels the course of the current is commonly described by two phases for which different terms are in use. In the first phase the current rises to a maximal value from which it continuously declines upon channel inactivation. We use here the term “transient current” which is otherweise also called “early” or “peak current”. The second phase is characterized by an almost very small current amplitude owing to the activity of non-inactivating channels. We use here the term “persistent current” which is identical to “late” or “plateau current”.

#### Statistics

If not directly stated by the presence of error bars, graphs show representative data from single cells. Average values from at least N = 5 cells are given as mean ± SD in the results section and in figure legends.

## Results

### TIC10 Blocks Human Na_v_1.5 Channels

**FIGURE 2:**
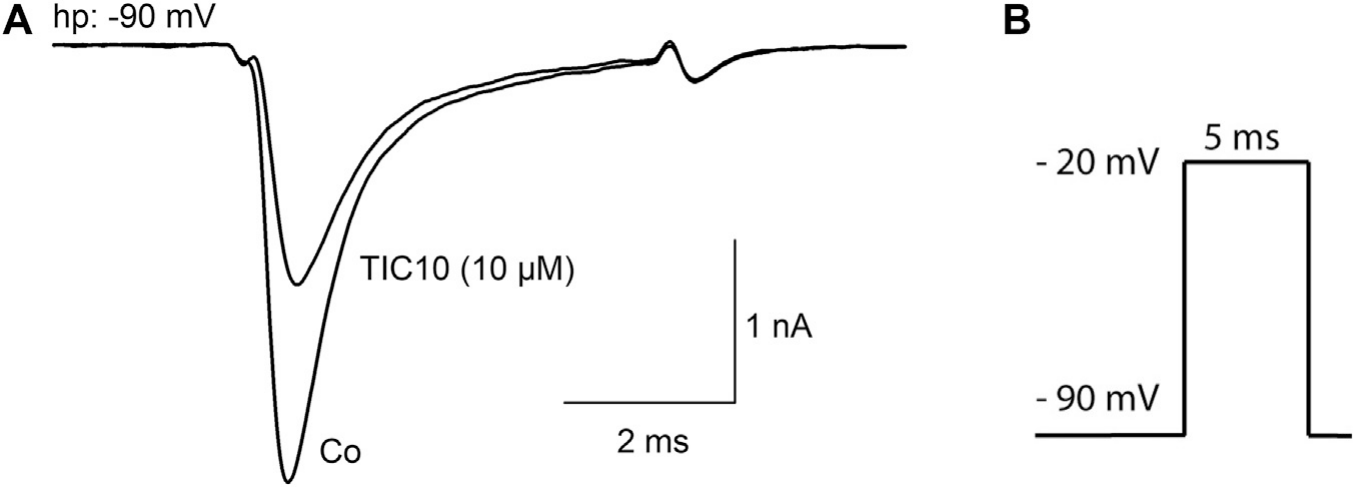
Inhibition of hNa_v_1.5 channels by TIC10. **(A)** Representative current traces obtained for control and in the presence of 10 µM TIC10. A similar outcome was obtained from four other cells. **(B)** Experimental scheme. Channel activations were carried out from a holding potential of −90 mV by depolarization to −20 mV for 5 ms.

The principal capability of TIC10 angular (TIC10) to block VGSCs is shown in Figure 2. To this end, channel activations in the absence and presence of 10 µM TIC10 were carried out by brief depolarizations to −20 mV using a holding potential of −90 mV, which was close to half-maximal inactivation in this experiment. Under these conditions, the current amplitude obtained for control was reduced to about half of its size in the presence of 10 µM TIC10 (Figure 2).

### Interaction With the Resting State

**FIGURE 3:**
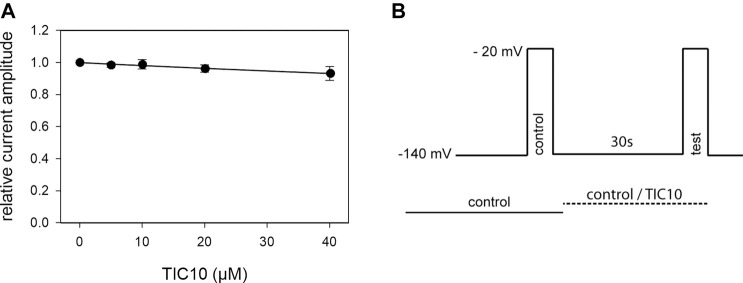
Block of resting hNa_v_1.5 channels. **(A)** Illustrated are relative current amplitudes in relation to the applied concentration of TIC10. Data points were fitted according to Eq. 1. Note, even at the highest concentration of TIC10 (40 µM), the inhibitory effect is far from being half-maximal. Thus, the calculated affinity (K_r_ = 600.4 ± 290.8 µM) has to be considered as a rough estimation. **(B)** Experimental scheme. Channel activations in the absence (control) and presence (test) of different concentrations of TIC10 were carried out from a holding potential of −140 mV. Current amplitudes obtained for the test-pulses were related to their controls and plotted against the concentration of TIC10.

As many sodium channel blockers exert their effects in a potential- or state-dependent manner, more specific investigations were undertaken, starting with the analysis for a probable interaction with the resting state. To this end, a double pulse protocol was conducted as illustrated in Figure 3B. Briefly, sodium channels were activated twice from a holding potential of −140 mV. The first activation was performed in the absence of drug and served as control. The second activation was elicited after a 30 s preincubation of control or TIC10 containing solution. For evaluation, current amplitudes in the presence of TIC10 were related to their respective controls and plotted against the concentration of TIC10 (Figure 3A). Of note, the inhibitory effect was far from being half-maximal. Even at the highest concentration tested (40 µM), the remaining current was still 93.2 ± 4.3% that of control. Therefore, a calculated affinity (K_r_) can only be considered as a rough estimation for the interaction of TIC10 with the resting state. According to Eq. 1, the half-maximal inhibition is calculated to occur at 600.4 ± 290.8 µM TIC10. Nevertheless, the inhibitory potency of TIC10 was strongly diminished when the channels were activated from a very negative holding potential (−140 mV) compared to an activation from a membrane potential in the range of half-maximal inactivation. Thus, it is obvious that the interaction of TIC10 strongly depends on the membrane potential.

### Voltage-Dependence of Activation

**FIGURE 4:**
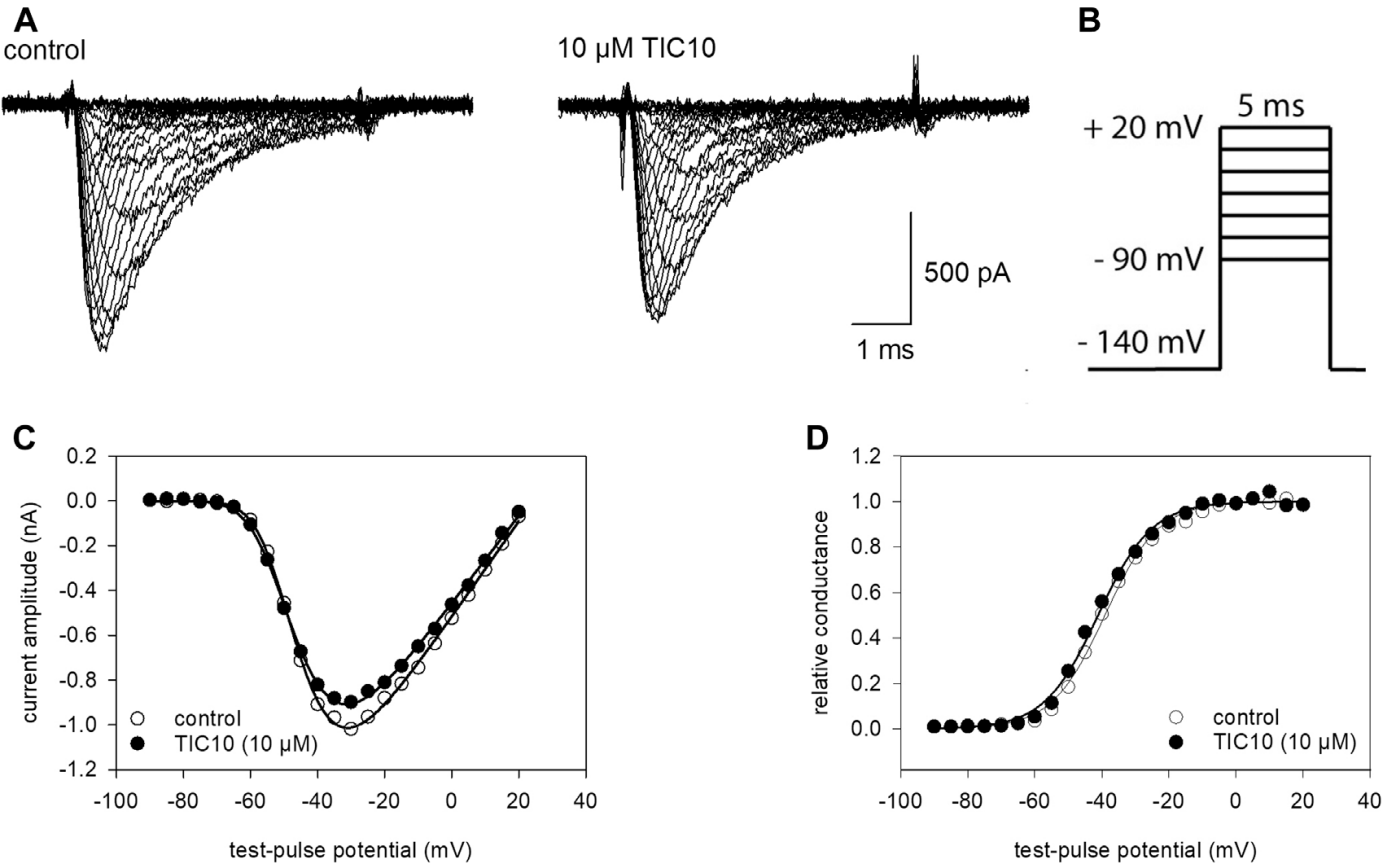
Voltage-dependence of activation. **(A)** Original traces obtained from channel activations in the absence (left) and presence of 10 µM TIC10 (right). **(B)** Experimental scheme: cells were clamped at a holding potential of −140 mV from which sodium currents were evoked by 5 ms test-pulses to potentials ranging from −90 to +20 mV (increment 5 mV). **(C)** Plots of maximal current amplitudes in the absence (open circles) and presence of 10 µM TIC10 (filled circles) versus the test potential. **(D)** Voltage-dependence of channel activation. Data from C were converted to conductance, normalized, and plotted versus the test-pulse potential. Half-maximal activation occurred at −43.1 ± 2.9 mV (k: 7.2 ± 0.6 mV) and −44.8 ± 2.8 mV (k: 7.9 ± 0.8 mV) for control and in the presence of 10 µM TIC10, respectively.

A next set of experiments was conducted to determine if TIC10 interacts with the process of channel activation. Here, brief depolarizations from a holding potential of −140 mV to different test-pulse potentials (range: −90 to +20 mV in steps of 5 mV) were carried out (Figure 4B). Original traces thereof are illustrated in Figure 4A. From these traces, the maximal current amplitude of each sweep was plotted against the applied test-pulse potential, resulting in a biphasic curve (Figure 4C). For further evaluation, current amplitudes were converted to their corresponding conductances respecting the reversal potential (Eq. 2). Next, normalized conductances for control and in the presence of TIC10 were plotted versus the test-pulse potential, whereby activation curves were obtained (Figure 4D). The potential of half-maximal activation and the slope of the curve were obtained from a fit of data using Eq. 3. If the inhibitory effect of TIC10 is based on an interaction with channel activation, a shift of the activation curve to more positive potentials is expected. However, this does not apply here. By contrast, TIC10 even provoked a minor shift of the activation curve to more negative potentials. With full details, midpoints of activation curves averaged for control at −43.1 ± 2.9 mV with a slope of 7.2 ± 0.6 mV. The corresponding values in the presence of TIC10 (10 µM) were −44.8 ± 2.8 mV with a slope of 7.9 ± 0.8 mV. Thus, the inhibitory impact of TIC10 on VGSCs does not arise from an interaction with the process of channel activation.

### Voltage-Dependence of Fast Inactivation

**FIGURE 5:**
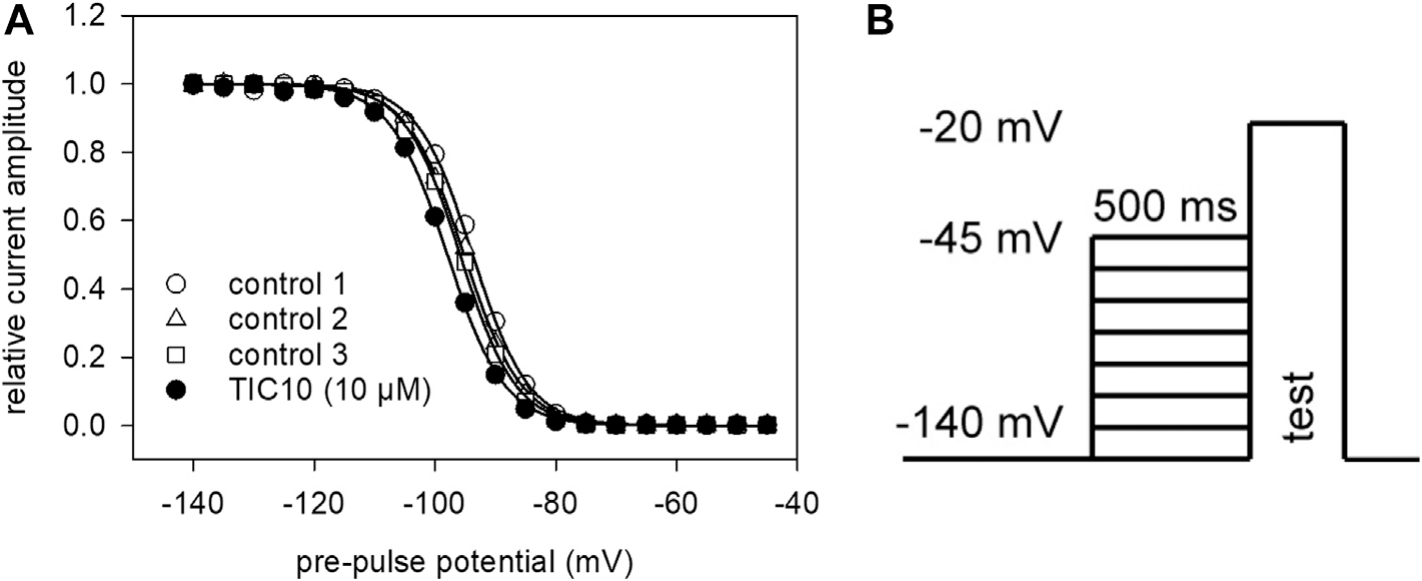
Voltage-dependence of fast inactivation. **(A)** Data of normalized transient currents in the absence (open symbols) and presence of TIC10 (filled circles) are plotted against the pre-pulse potential. Solid lines represent fits according to Eq. 4. Respecting the drug independent shift, inactivation midpoints shifted in the presence of 10 µM TIC10 by 1.2 ± 1.4 mV to more negative potentials. **(B)** Experimental scheme. Voltage-dependence of fast inactivation was determined by measuring sodium currents elicited by 5 ms depolarizations to −20 mV after conditioning pre-pulses for 500 ms to different potentials (range −140 to −45 mV).

The aim of the following experiment was to see if and how TIC10 interacts with the process of fast inactivation. To vary the amount of inactivated channels, pre-pulses of constant duration (500 ms) with an increasing pre-pulse potential (range −140 to −45 mV) were applied immediately before the test-pulses (Figure 5B). Thereby, the number of inactivated channels increased as a function of the pre-pulse potential. For evaluation, all current amplitudes were related to the amplitude obtained from the most negative pre-pulse potential (−140 mV). The relative current amplitudes were then plotted versus the pre-pulse potential. Data were fitted according to Eq. 4 in order to generate inactivation curves. The most important parameters here are the potential at which half of the channels are inactivated and the slope of the curve. If the inhibitory action of TIC10 is based on an interaction with fast inactivation a shift of the inactivation curve to more negative potentials is expected. As inactivation curves also undergo a drug independent left shift, mimicking an inhibitory effect, this shift was separately estimated in individual cells and taken into account in the evaluation of drug effects. The outcome of a typical experiment is illustrated by Figure 5A. Altogether, for 10 µM TIC a shift of −1.2 ± 1.4 mV was calculated. The slope was 5.3 ± 0.9 mV in the absence and 5.4 ± 0.9 mV in the presence of 10 µM TIC10. These data indicate that TIC10 exerted no or only a minimal effect on fast inactivation.

### Interaction With the Slow Inactivated State

**FIGURE 6:**
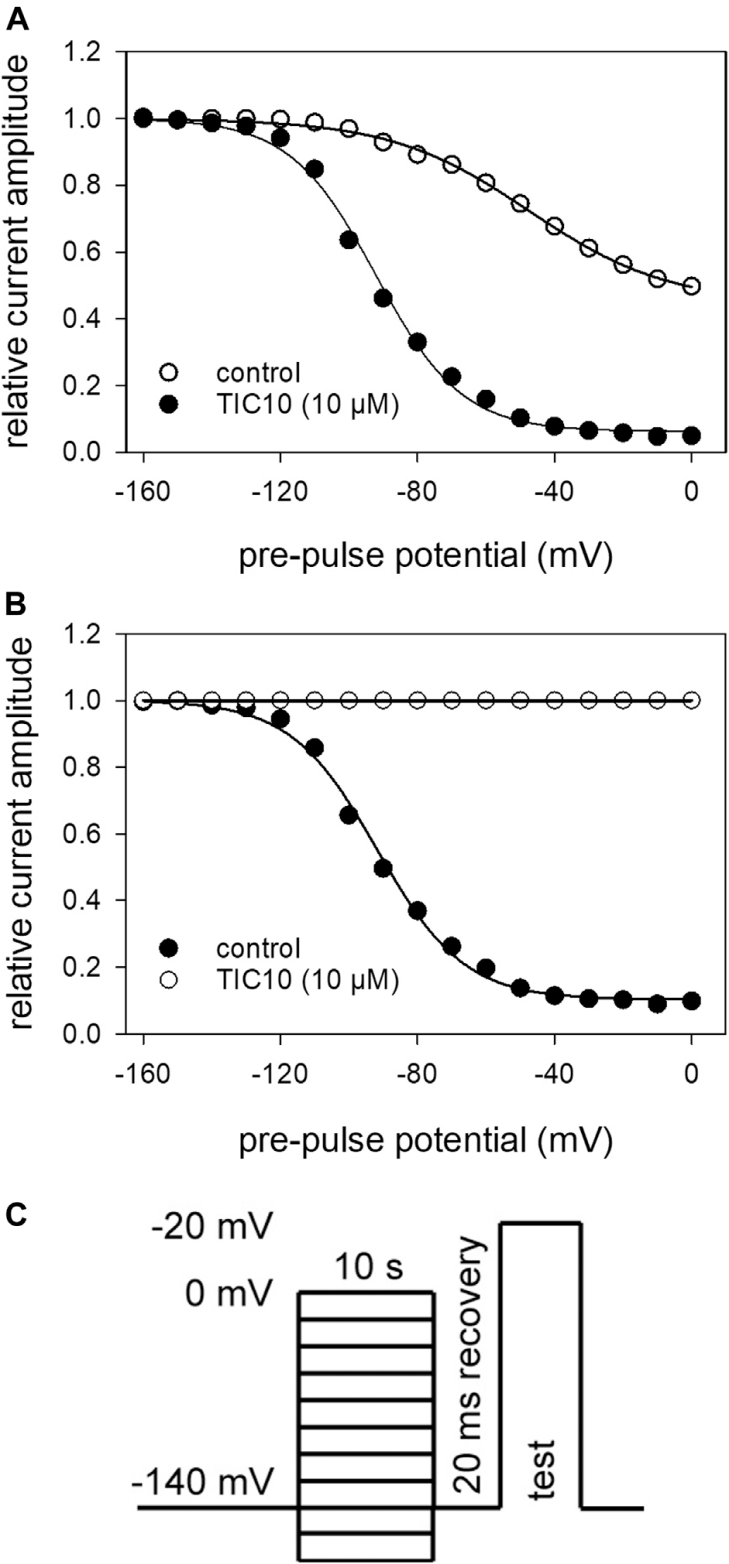
Voltage-dependence of slow inactivation. **(A)** Relative current amplitudes in dependence of the pre-pulse potential. Solid lines are fits according to Eq. 5 for control (open circles) and TIC10 (10 μM, filled circles). **(B)** Normalized data with resepct to control. **(C)** Experimental scheme. Activations to −20 mV were elicited after long-lasting (10 s) conditioning pre-pulses to different potentials (range: −160 to 0 mV; increment 10 mV) and a short (20 ms) recovery period at −140 mV immediately before the test-pulse.

The protocol for analyzing an interaction with the slow inactivated state differed in two points from that of fast inactivation. First, the duration of the pre-pulse was prolonged to 10 s. Second, immediately before the test-pulse a short recovery period (20 ms, −140 mV) was inserted to eliminate or minimize the contribution from fast inactivation (see Figure 6C). Under control conditions, the current amplitudes become smaller at pre-pulse potentials positive to around −100 mV. Half-maximal inactivation occurred at −42.9 ± 5.0 mV with a slope of 21.5 ± 2.7 mV. At a pre-pulse potential of 0 mV, the remaining current amplitude amounted to 45.6 ± 10.9%. In the presence of TIC10, the slow inactivation curve was shifted to more negative values. In particular, half-maximal inactivation occurred at −92.7 ± 7.0 mV with a slope of 12.8 ± 0.9 mV. Furthermore, availability at 0 mV dropped to 4.7 ± 2.8% (Figure 6A). In order to ascribe this behavior either to an interaction with the fast or slow inactivated state, the potential-dependent behavior was inspected in more detail. As even under control, a clear potential dependency was obvious, TIC10-induced changes were normalized with respect to control by dividing drug values by control values (Figure 6B). If the inhibition is related to an interaction with the slow inactivated state, inhibition is expected to increase in the potential range where slow inactivation is likely to occur, i.e. in a potential range positive to that of fast inactivation. It turned out that the inhibition did not increase significantly in the range between −40 and 0 mV. In particular, inhibition amounted to 88.3 ± 5.0% and 89.7 ± 6.5%, respectively. Taken together, these findings did not argue for an interaction with the slow inactivated state. Nevertheless, the interaction of TIC10 with the hNa_v_1.5 occurred on a slow time course.

### Time Dependence of Interaction

**FIGURE 7:**
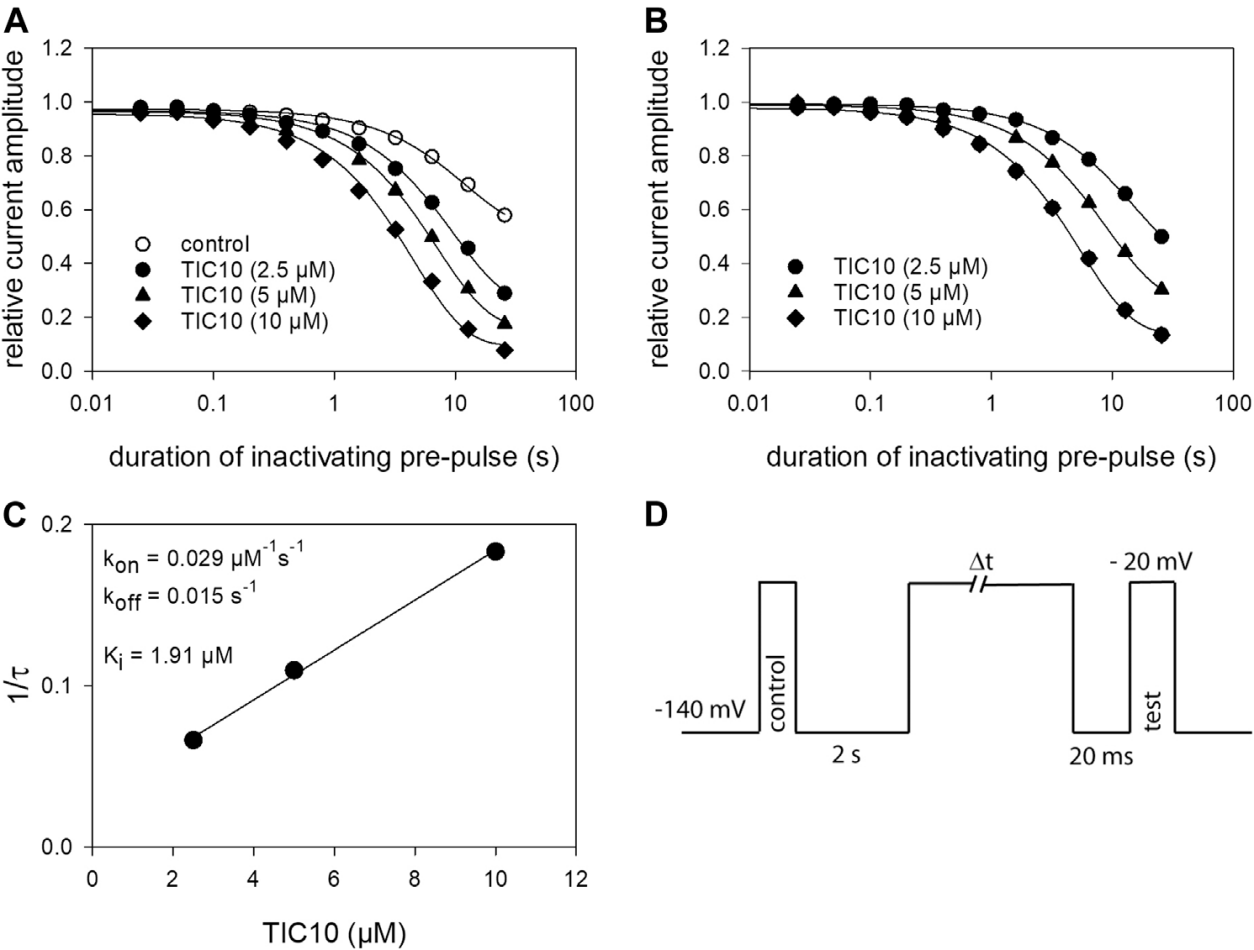
Time-dependent interaction. **(A)** The graph illustrates relative current amplitudes in the absence and presence of TIC 10 versus the duration of an inactivating pre-pulse to −20 mV. Solid lines describe individual time courses obtained in the absence and presence of TIC10 using single exponential functions for data fitting. **(B)** Data from A, normalized with respect to control. **(C)** Plot of the inverse of the time constants estimated from the data as illustrated in B versus the concentration of TIC10. K_on_ and k_off_ values were taken from the slope and the *y*-intercept of the linear regression, respectively. The affinity for the inactivated state (K_i_) was calculated according to Eq. 10 to be 1.91 µM in this experiment. Altogether a K_i_ of von 1.86 ± 0.7 µM resulted. **(D)** Experimental scheme. Starting from a holding potential of −140 mV, first a control pulse to −20 s was executed. Thereafter, ample time (2 s) was given to allow for full recovery before channels were inactivated for a variable duration at −20 mV. The test-pulse was carried out after a short recovery at −140 mV. For evaluation, test-pulse amplitudes were related to their controls.

Next, a set of experiments was performed to describe the time course of block development. The protocol is illustrated in Figure 7D. Briefly, after channel activation from a holding potential of −140 mV for control, ample time for full recovery at the holding potential was provided. Thereafter, the channels were inactivated for a variable duration (range: 25 ms–30 s) at a potential of −20 mV. Immediately before the test-pulse, channels were allowed to recover once more at the holding potential to eliminate or minimize the impact from fast inactivation. For evaluation, the current amplitudes obtained for test-pulses were related to their controls. Using this protocol in the absence of TIC10, relevant reductions of availability were observed at inactivation times lasting longer than 1 s. The estimated time constant was 10.1 ± 2.0 s. At the longest inactivation time, the current amplitude dropped to 59.2 ± 7.7%. In the presence of TIC10, the availability already decreased at shorter inactivation times with a much more pronounced reduction at the longest inactivation time (Figure 7A). In particular, for 2.5 µM TIC10 a time constant of 5.3 ± 1.9 s with a remaining current of 46.0 ± 4.8% was estimated. The corresponding values for 10 µM TIC10 were 2.0 ± 0.8 s and 16.9 ± 6.4%. For further evaluation, data were normalized with respect to control values (Figure 7B). To obtain time constants for block development, data were fitted with single exponential functions. Afterwards, the inverse, the inverse of the time constants was plotted versus the concentration of TIC10 (Figure 7C). The on-rate (*k*_*on*_) was directly taken from the slope of the linear regression and the off-rate (*k*_*off*_) was given by the *y*-intercept (Eq. 9, 10). For estimating the affinity for the inactivated state, the *K*_*i*_ values were separately calculated for each cell according to the relation *K*_*i*_ *= k*_*off*_ */k*_*on*_. The values for one representative cell are given as insert to Figure 7C. Overall, a K_i_ of 1.86 ± 0.7 µM was determined.

### Interaction With the Inactivated State: Steady-State Parameters

**FIGURE 8:**
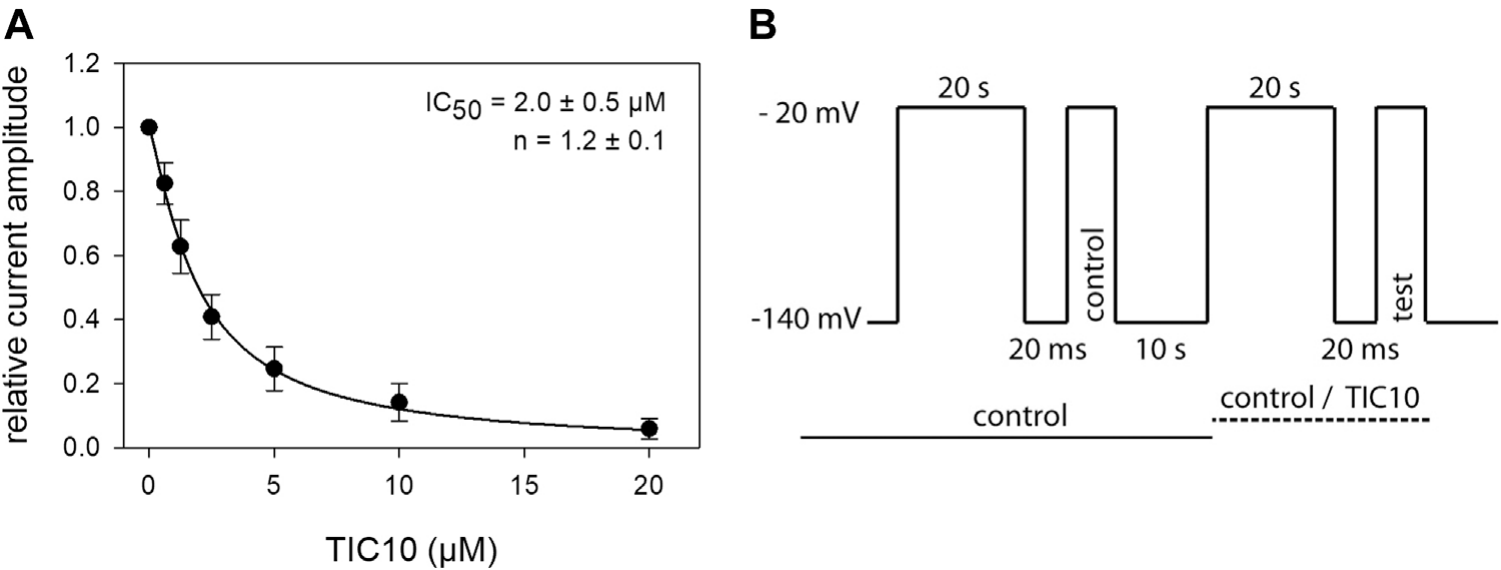
Interaction with the inactivated state: steady-state parameters. **(A)** The graph shows relative current amplitudes upon channel activations in the presence of different concentrations of TIC10, after a prolonged inactivation. **(B)** Experimental scheme, illustrating the double pulse protocol. Starting from a holding potential of −140 mV channels were inactivated for 20 s at a potential of −20 mV. After a short recovery at holding potential, the control pulse was executed. Between the control and test run (second part), ample time (10 s) for full recovery was provided. In the second part, the protocol was repeated in either control or test solution. For evaluation, test current amplitudes were related to their controls. Data analysis was restricted to data sets where control and test-pulse amplitude in mere control solution did not deviate by more than 3%. Overall, half-maximal inhibition (IC_50_), representing the affinity for the inactivated state (K_i_) was calculated to be 2.0 ± 0.5 µM.

As TIC10 revealed strong effects after prolonged inactivation, additional experiments were conducted under these conditions to establish a full concentration-response relationship. To this end, a double pulse protocol was used as outlined by Figure 8B. Briefly, channel activations for control and in the presence of different concentrations of TIC10 were carried out after channels had been inactivated for 20 s at a potential of −20 mV. To minimize drift effects during these long-lasting experiments, each run included its own control. Thus, drug effects as measured by the test-pulse were related to the timely executed control activation. Data analysis was restricted to cells where the current amplitude due to control- and test-pulse in the presence of mere control solution did not deviate by more than 3%. Under these conditions, an affinity for the inactivated state with a K_i_ of 2.0 ± 0.5 µM was calculated (Figure 8A). Interestingly, the K_i_ values estimated from kinetic data (see previous section) and steady-state parameters are identical.

### Interaction With Partially Inactivated Channels

**FIGURE 9:**
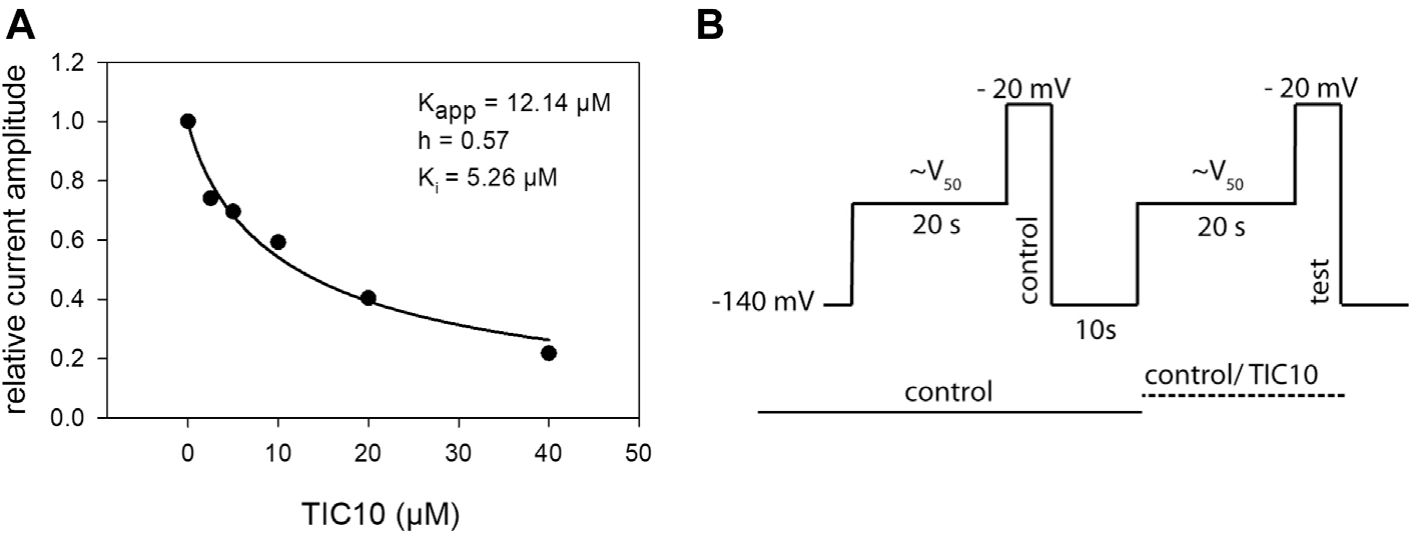
Interaction with partially inactivated channels. **(A)** Illustrated are relative currents amplitudes in relation to the applied concentration of TIC10 upon partial inactivation of the channels of one representative cell (out of five experiments). For evaluation data were fitted with Eq. 1, giving K_app_. In order to estimate the affinity for the inactivated state (K_i_), the current amount of non-inactivated channels (h) was considered according to Eq. 7, resulting in a mean K_i_ of 4.7 ± 1.3 µM (N = 5). Data evaluation (relation of test-pulse to control pulse) was restricted to experiments where the current amplitudes obtained from mere control solution did not deviate by more than 3%. **(B)** Experimental scheme. From a holding potential of −140 mV conditioning pre-pulses to a potential of about half-maximal inactivation (V_50_) were applied for 20 s before the test-pulse to −20 mV was executed. This protocol was executed a second time, either in the presence of control solution or different concentrations of TIC10.

In order to reflect more the physiological situation, concentration-relationships were performed at a presumed resting membrane potential of −90 mV were the channels exist in the fast and slow as well as in the resting state. Again, the experimental scheme consisted of a double pulse protocol that deviated from the previous one by the potential for inactivation and the lack of a recovery period before the control- or test-pulse (Figure 9B). In any case, the inactivation potential was estimated for each cell individually from another double pulse protocol, which was restricted to the relevant part of the inactivation curve. For evaluation, the current amplitudes obtained for the test-pulses were related to their controls and plotted versus the concentration of TIC10 (Figure 9A). Data points were fitted with Eq. 1, whereby so-called apparent affinity constants (K_app_) resulted. The word “apparent” is used to indicate that the resulting affinity constant is composed from the affinity to the resting and the affinity to the inactivated state. In order to recalculate the affinity for the inactivated state, Eq. 7 was used. This requires the affinity for the resting state (600 µM), obtained from previous experiments, and the current amount of inactivated channels, which was estimated in individual cells immediately before starting the experiments. As the current amount of inactivated channels varies between individual cells, each cell was evaluated separately. Altogether, an affinity to the inactivated state of 4.7 ± 1.3 µM resulted. Of note, the affinity for the inactivated state estimated here is less than that obtained from the other experiments.

### Recovery From Inactivation

**FIGURE 10:**
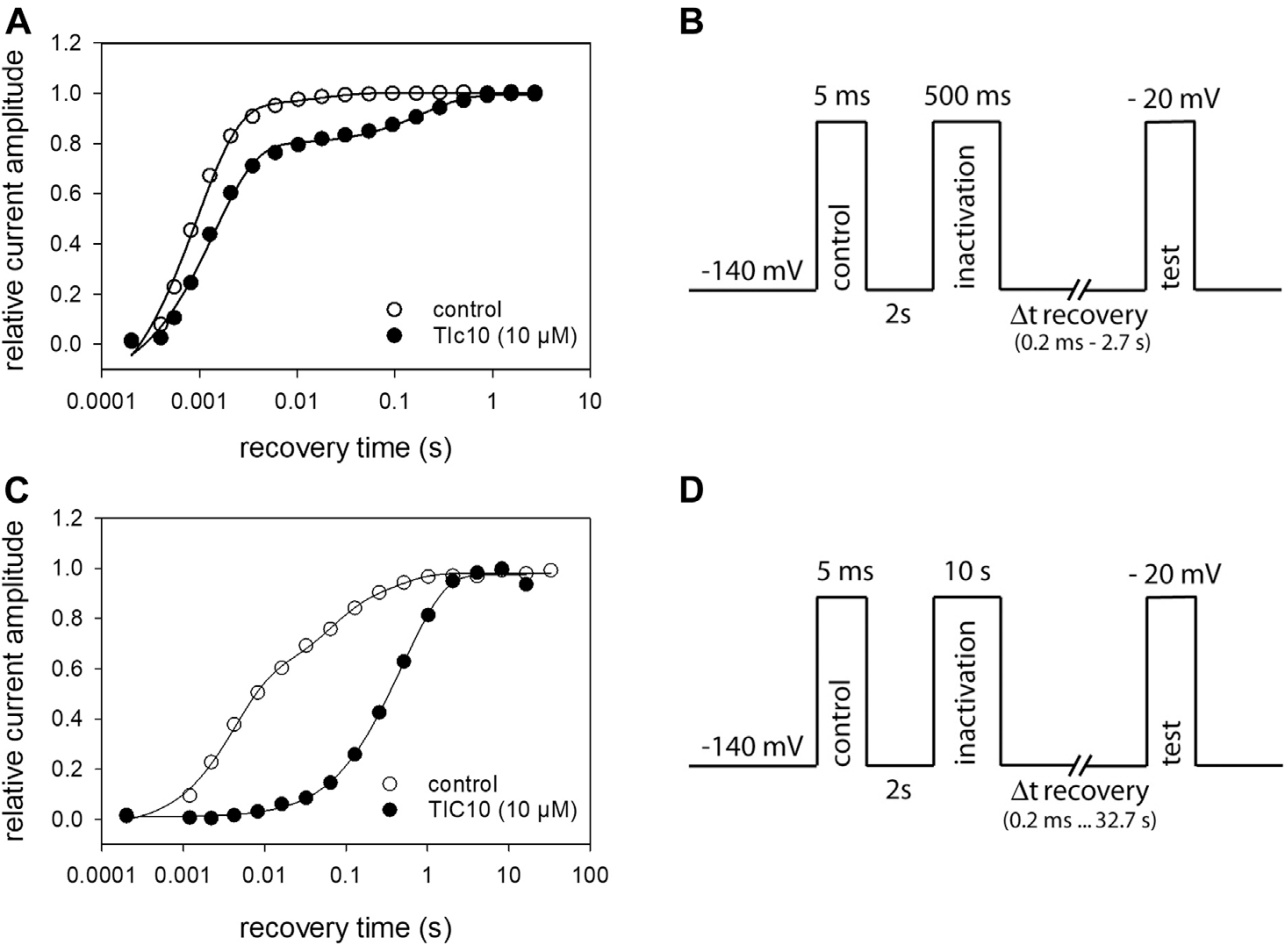
Recovery from inactivation. **(A)** Recovery from fast inactivation. The relative amount of available channels versus the recovery time at −140 mV is illustrated. Solid lines represent fits with two exponential functions according to Eq. 11. **(B)** Experimental scheme. After an initial control pulse, inactivation was performed at −20 mV for 500 ms. The test-pulse was applied after a varying time of recovery. For evaluation, current amplitudes of the test-pulses were related to their controls. Interval between individual runs was 10 s and TIC10 was applied at 10 µM. **(C)** Recovery from slow inactivation. In case of control, three exponentials were required to describe the time course of recovery; in the presence of TIC10, two exponentials were sufficient. **(D)** Experimental scheme for recovery from slow inactivation. Otherwise, identical protocol as in **(A)** but with the inactivation time set to 10 s and recovery time prolonged up to 32.8 s.

If a drug interacts with the inactivated state (fast or slow), it is expected that also recovery from inactivation is affected. We analyzed this aspect by the use of a tri-pulse experiment (Figure 10B). After an initial control pulse, sufficient time was provided for full recovery. Next channels were inactivated for 500 ms at −20 mV. The test-pulse was initiated after channels had been allowed to recover at a potential of −140 mV for a variable time (range: 0.2 ms to 2.7 s). For evaluation, the current amplitudes of the test-pulses were related to their controls and plotted versus the recovery time (Figure 10A). To describe the time course of recovery, data points were fitted with double exponential functions (Eq. 11). Under control condition, most of the channels (92.6 ± 4.0%) recovered with a time constant of τ_1_ = 1.4 ± 0.6 ms, the remainder with τ_2_ = 23.6 ± 12.0 ms. In the presence of 10 µM TIC10, the proportion of fast recovering channels decreased to 75.6 ± 7.0% while the recovery time slightly increased to 2.3 ± 1.2 ms. The most prominent effect of TIC10 here was the about tenfold increase of the second time constant to 267.6 ± 73.1 ms. In order to estimate recovery from slow inactivation, the experimental scheme was modified to a prolonged inactivation time (10 s) and an expansion of the recovery time up to 32.8 s (Figure 10D). As expected, the effect of TIC10 was more pronounced here (Figure 10C). Under control, three exponentials were required to describe the time course of recovery, whereas in case of TIC10, two time constants were sufficient (Table 1).

**TABLE 1:** Time constants for recovery from slow-inactivation of TIC10 (10 µM).

	Control	Amount	TIC10	Amount
τ_1_ (ms)	4.8 ± 4.9	53.3 ± 5.2%	128.1 ± 80.3	36.3 ± 16.0%
τ_2_ (ms)	54.4 ± 45.2	31.0 ± 4.5%	548.4 ± 287.4	62.2 ± 15.7%
τ_3_ (ms)	486.9 ± 389.0	14.8 ± 2.9%	-	-

### Tests for an Open Channel Blocking Mechanism—Interaction With the Persistent Current.

**FIGURE 11:**
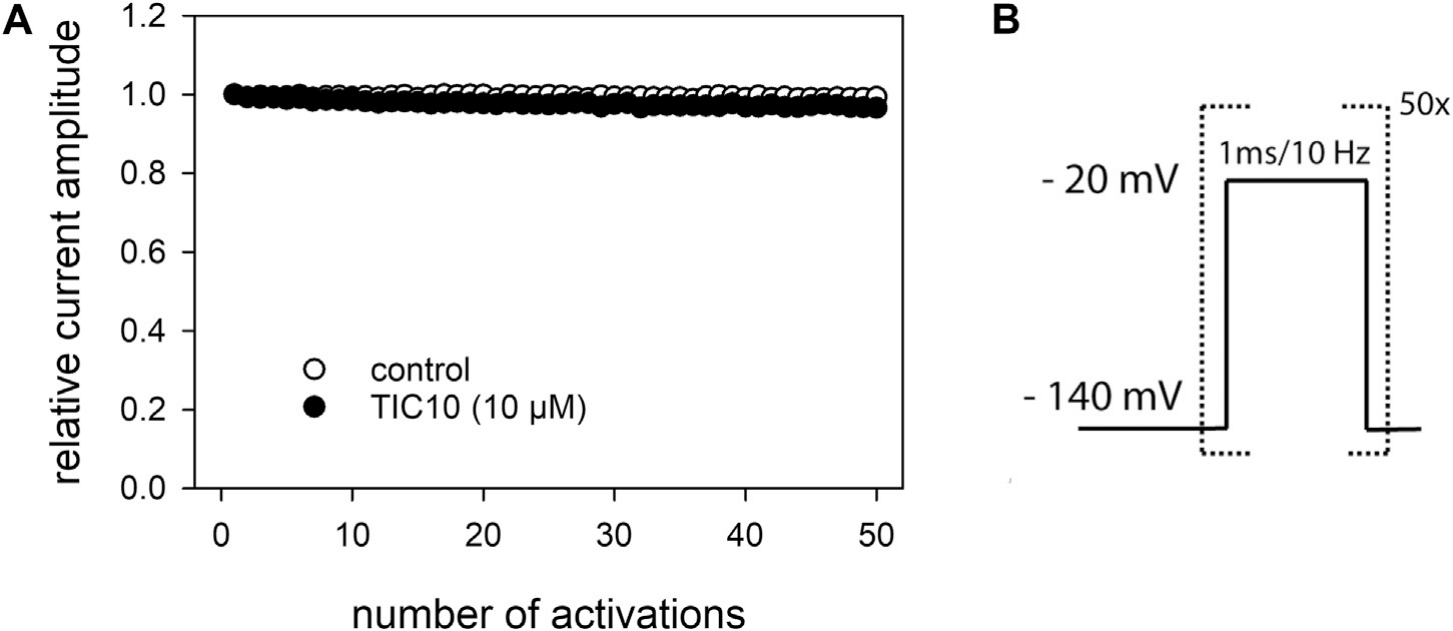
Analysis for use-dependence. **(A)** Illustrated is the relative current amplitude versus the number of channel activations. **(B)** Experimental scheme. Channel activations were carried out by brief pulses (1 ms) to −20 mV at a frequency of 10 Hz in the absence and presence of TIC10 (10 µM). Current amplitudes minimally decline under control from the first to the last activation (50th pulse) to 99.5 ± 0.6% and in the presence of TIC10 (10 µM) to 95.7 ± 1.6%. TIC10 was preincubated for 20 s before starting the high frequency activation.

There are several protocols in use to test for an open channel blocking mechanism. In this work, two of them were used: Use-dependency and inactivation-deficient mutants. In a first set of experiments, we looked for a use-dependent behavior. To this end, short test-pulses (1 ms) of −20 mV were applied from a holding potential of −140 mV (Figure 11B). We fixed on this short activation time in order to minimize the inactivation time during each activation cycle. Prior to the high frequency stimulation, infrequent activations were carried out (0.2 Hz) until a stable baseline was achieved. Thereafter, either control solution or TIC10 was perfused for 20 s before the high frequency stimulation (10 Hz) was started. For evaluation, the current amplitude of each pulse was divided by the amplitude of the first response of the high frequency stimulation train. For control, current amplitude minimally declined to 99.5 ± 0.6%. In the presence of TIC10, the remaining current of the last activation amounted to 95.7 ± 1.6% (Figure 11A). Thus, TIC10 revealed no or only a minimal use-dependent behavior.

**FIGURE 12:**
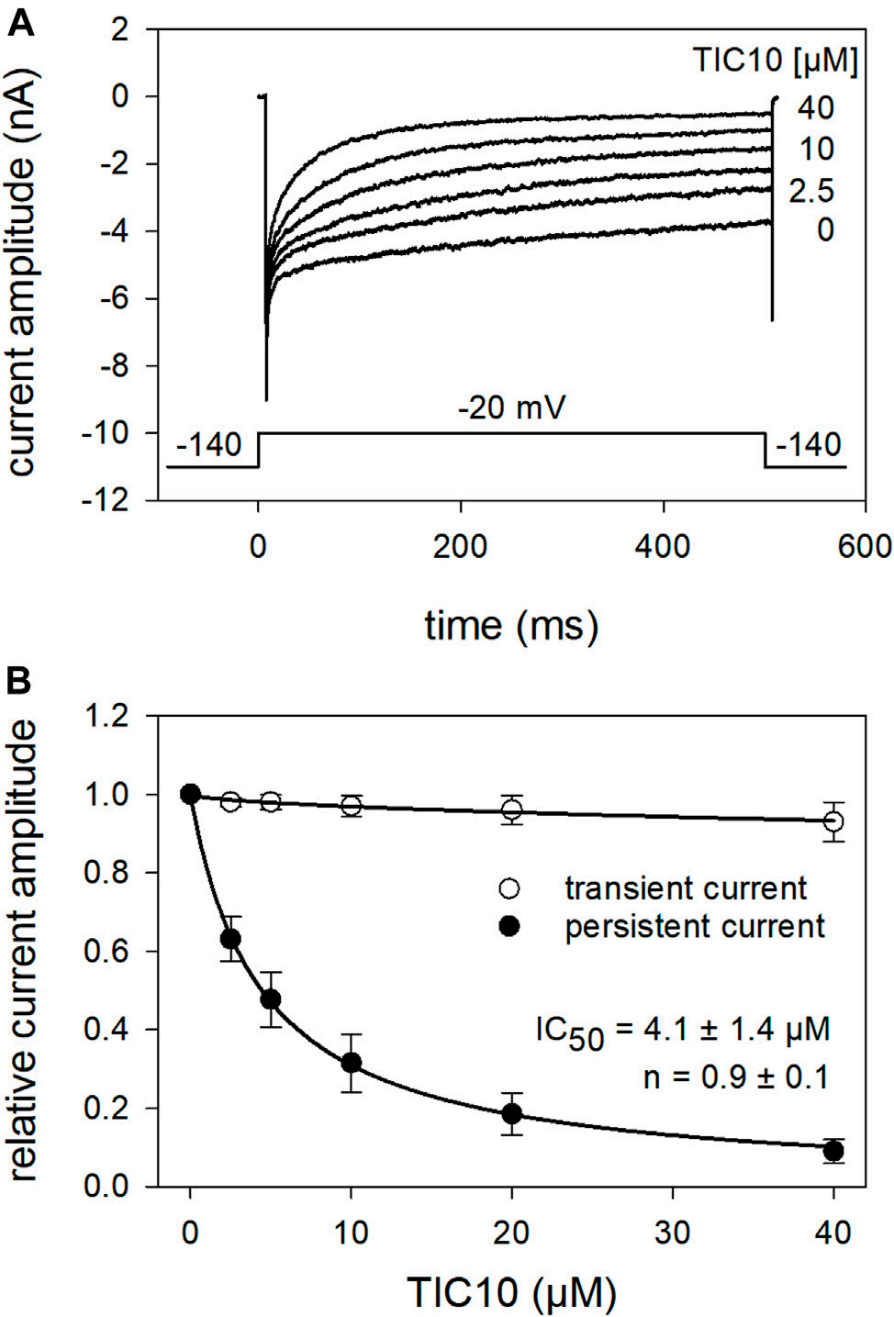
Interaction of TIC 10 with the open state. The possible interaction with the open state was analyzed by means of the WCW mutant. **(A)** Original current traces obtained in the absence and presence of different concentrations of TIC10. Insert: experimental scheme. Cells were held at a holding potential of −140 mV. Individual activations were carried out by test-pulses to −20 mV for 500 ms after a preincubation of TIC10 for 20 s. The transient current was hardly affected while the persistent current, measured at the end of the test-pulse, was reduced in a concentration dependent manner. **(B)** Relative transient and persistent current amplitudes in relation to the concentration of TIC10.

In the next experiments, we tested a channel mutant with reduced capability to inactivate (hNa_v_1.5_I408W_L409C_A410W; WCW mutant). With this mutant, the channels stay open to a large extent even after a prolonged activation (500 ms), as evidenced by a persistent current. If a drug leads to a concentration-dependent reduction of this persistent current, it can also be regarded as an interaction with the open state. Original traces from these experiments are illustrated in Figure 12A. It is evident that the tranisent current, representing drug binding to the resting state, is hardly affected in the presence of TIC10 up to a concentration of 40 µM (Figure 12B). Thus, no estimation of the half-maximal effective concentration was performed. By contrast, the persistent current was strongly diminished in a concentration-dependent manner with a half-maximal inhibition occurring at 4.1 ± 1.4 µM TIC10 with a Hill-coefficient of 0.9 ± 0.1. Thus, TIC10 also operated as an open channel blocker.

### Identification of the Possible Interaction Site by Channel Mutants

**FIGURE 13:**
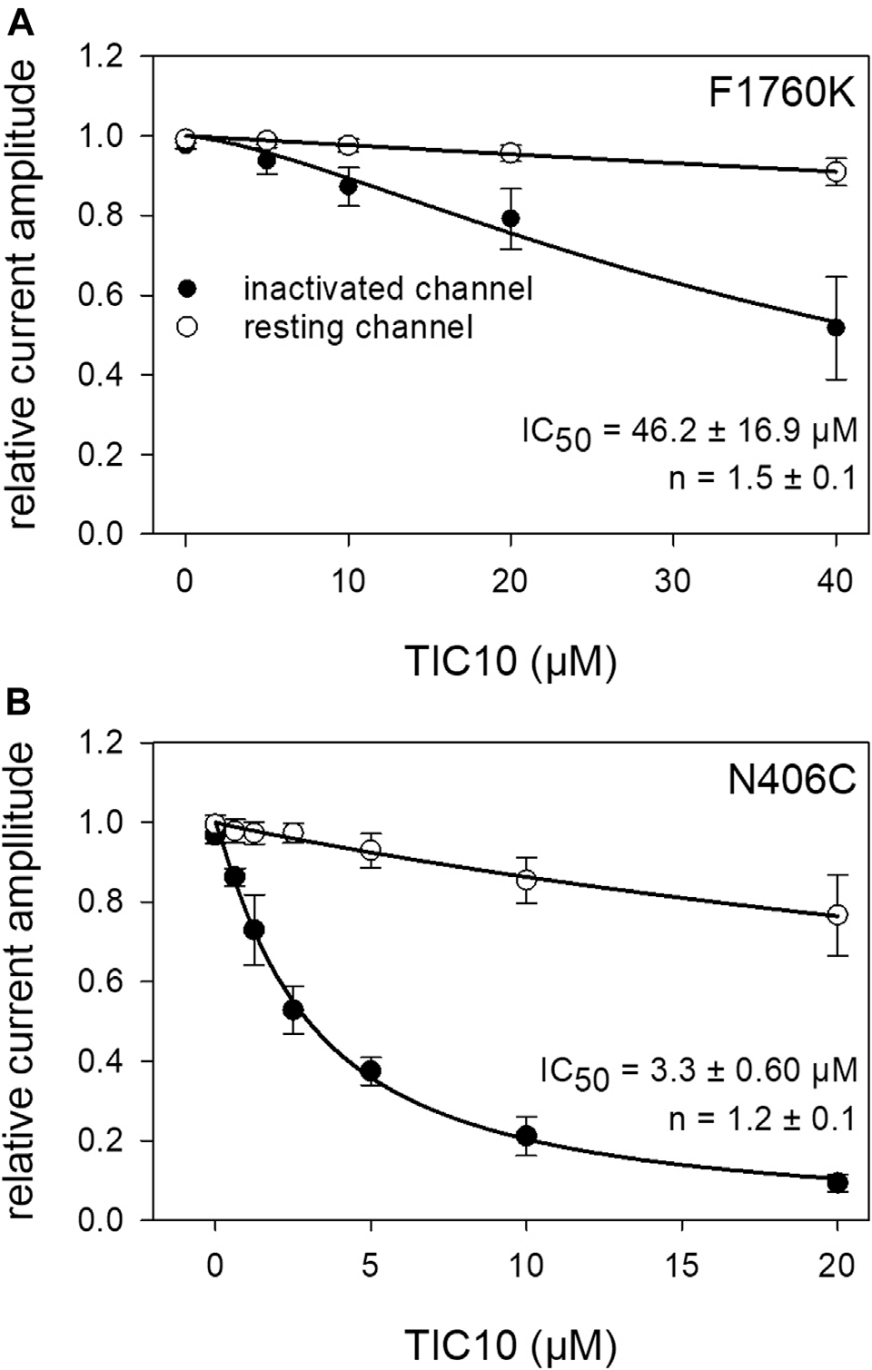
Interaction with receptor mutants. **(A)** Concentration-response curve obtained with the F1760K mutant. Relative current amplitudes for resting and inactivated channels are plotted versus the concentration of TIC10. Due to the small effect at resting channels, no effective concentration was estimated. In case of inactivated channels half-maximal inhibition was calculated to be 46.2 ± 16.9 µM. Experiments for the resting and inactivated state were performed as illustrated by Figures 3, 8, respectively. **(B)** Concentration-response curve for the N406C mutant. Identical experiments as outlined in A, carried out with the mutant N406C. Half-maximal effects for inactivated channels were obtained at 3.3 ± 0.6 µM TIC10.

To look for a possible interaction site at the hNa_v_1.5, we used two mutants, F1760K and N406C. The F1760K mutant is one out of several mutants, which affects the local anesthetic binding site (Catterall, 2014). This site is not only important for local anesthetics but also for many blockers of VGSCs. If a drug primarily interacts at the position 1760, a strongly reduced affinity is expected in case of the F1760K mutant. This clearly applied for TIC10. The estimated affinity for the slow inactivated channels was 46.2 ± 16.9 µM (Hill-coefficient: 1.5 ± 0.1), which is more than 20-fold less affine compared to the wild type (Figure 13A). Another important interaction site which affects the affinity for local anesthetics is N406. This site additionally has an impact on slow inactivation (McNulty et al., 2006). Using the N406C mutant, the affinity for TIC10 marginally changed compared to the wild type (Figure 13B). In particular, the estimated IC_50_ was 3.3 ± 0.6 µM with a Hill-coefficient of 1.2 ± 0.1.

### Investigation of Imipridone Derivatives

**FIGURE 14:**
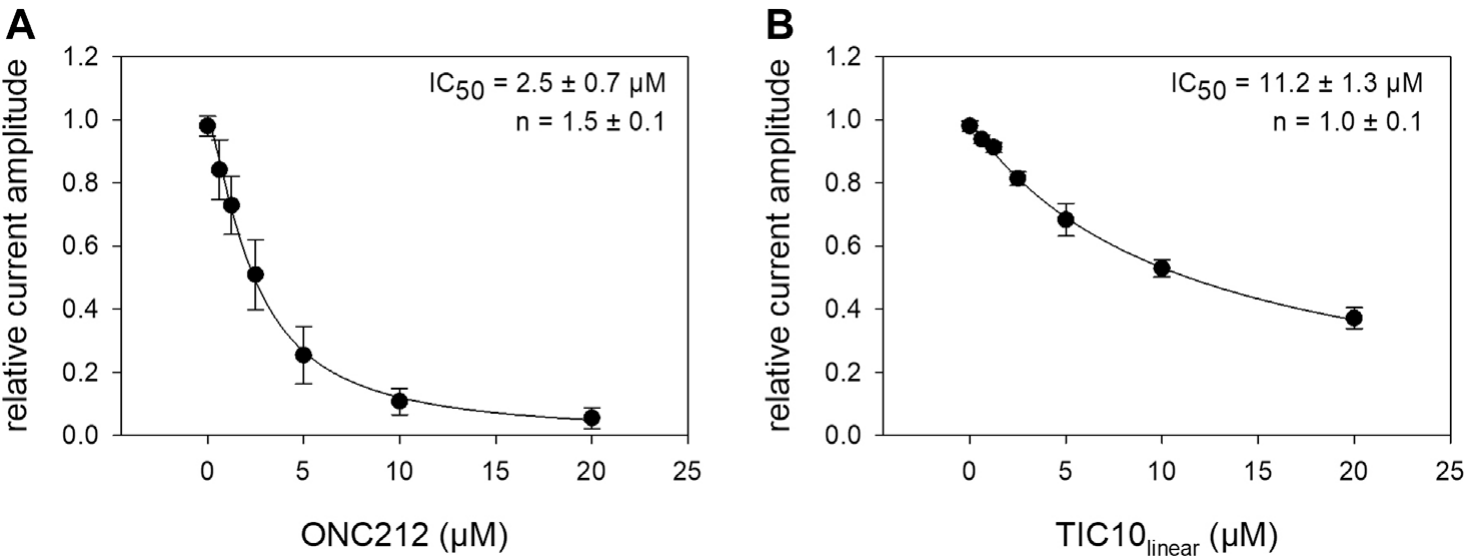
Interaction of imipridone derivatives with the inactivated state. The graphs show relative current amplitudes upon channel activations in the presence of different concentrations of imipridone derivates after a prolonged inactivation (20 s). Experimental scheme as illustrated by Figure 8B. **(A)**. Data for ONC212, revealed a half-maximal inhibition at 2.5 ± 0.7 µM. **(B)** Data for TIC10_linear_ with a half-maximal effect at 11.2 ± 1.3 µM.

**FIGURE 15:**
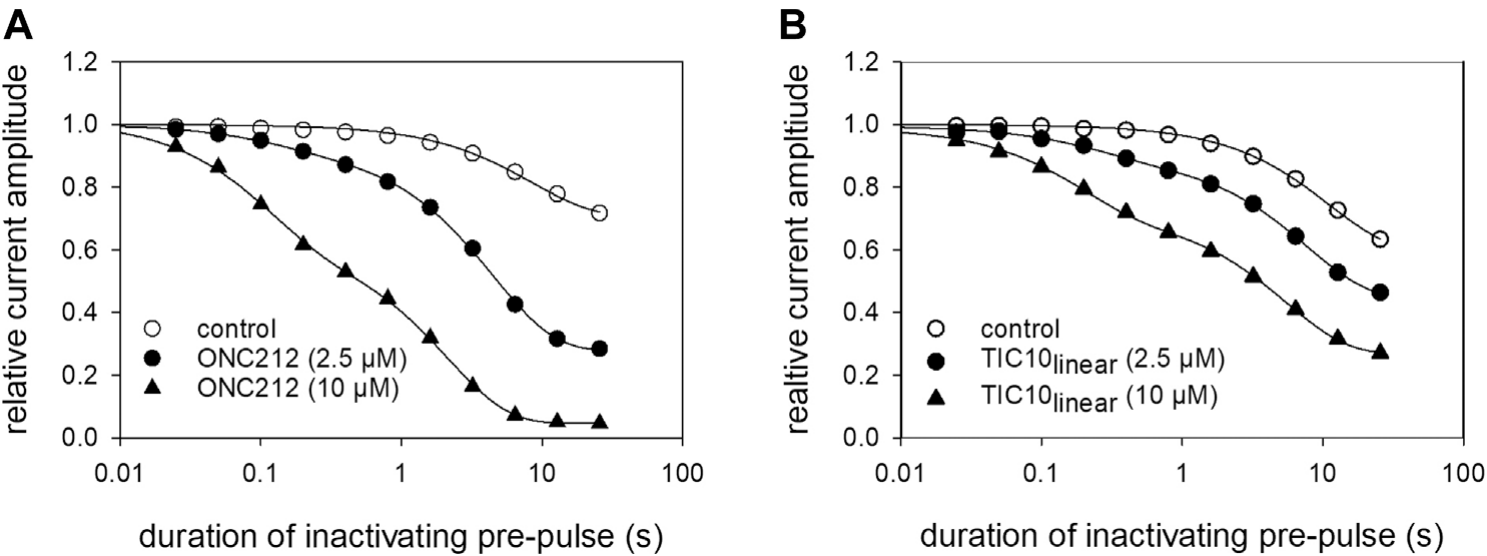
Time-dependent interaction of imipridone derivatives. The graph illustrates relative current amplitudes in the absence and presence of different concentrations of **(A)** ONC212 and **(B)** TIC10_linear_ versus the duration of the inactivating pre-pulse to −20 mV. Solid lines describe individual time courses obtained in the absence and presence of TIC10 using double exponential functions for data fitting. Experimental scheme as illustrated by Figure 7D. Details of evaluation are given by Table 2.

**FIGURE 16:**
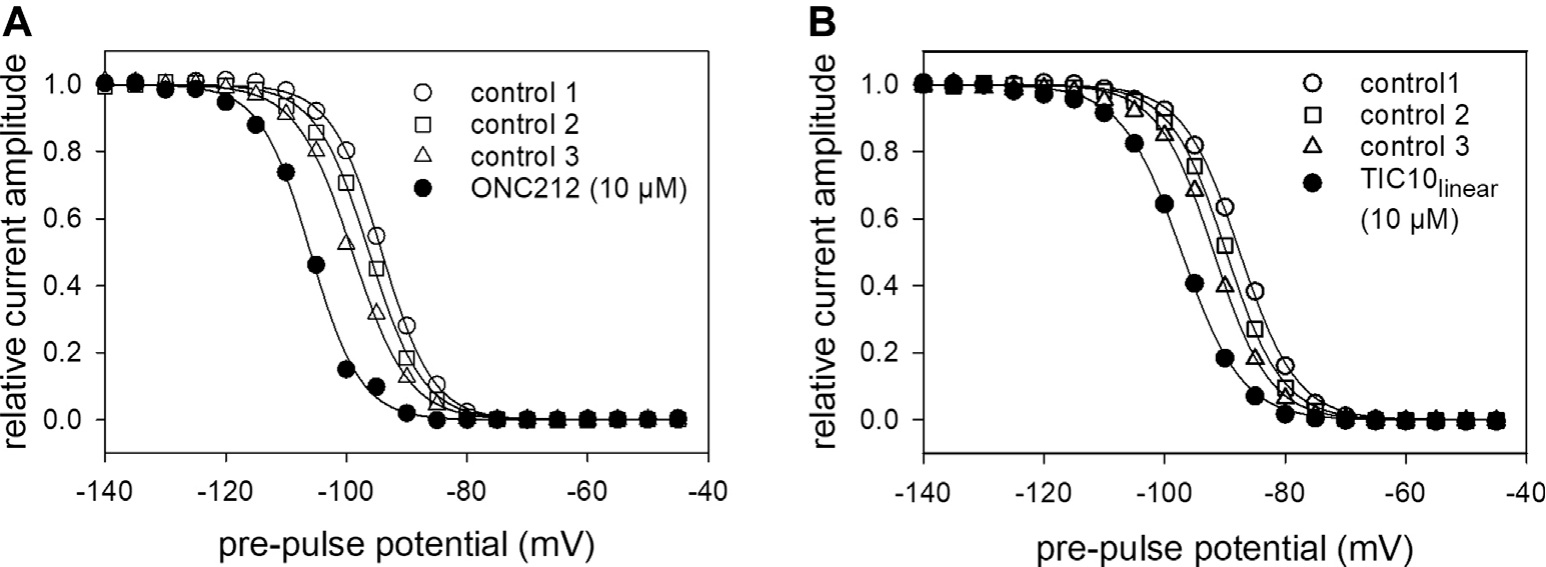
Impact of imipridones derivatives on fast inactivation. Data of normalized transient currents in the absence (open symbols) and presence of drug (filled circles) are plotted against the pre-pulse potential. Experimental scheme as in Figure 5B. **(A)** ONC212 shifted inactivation midpoints by −4.7 ± 0.42 mV. **(B)** TIC10_linear_ shifted inactivation midpoints by −3.0 ± 0.37 mV. Data are corrected for the endogenous shift, estimated from three preceding controls.

**FIGURE 17:**
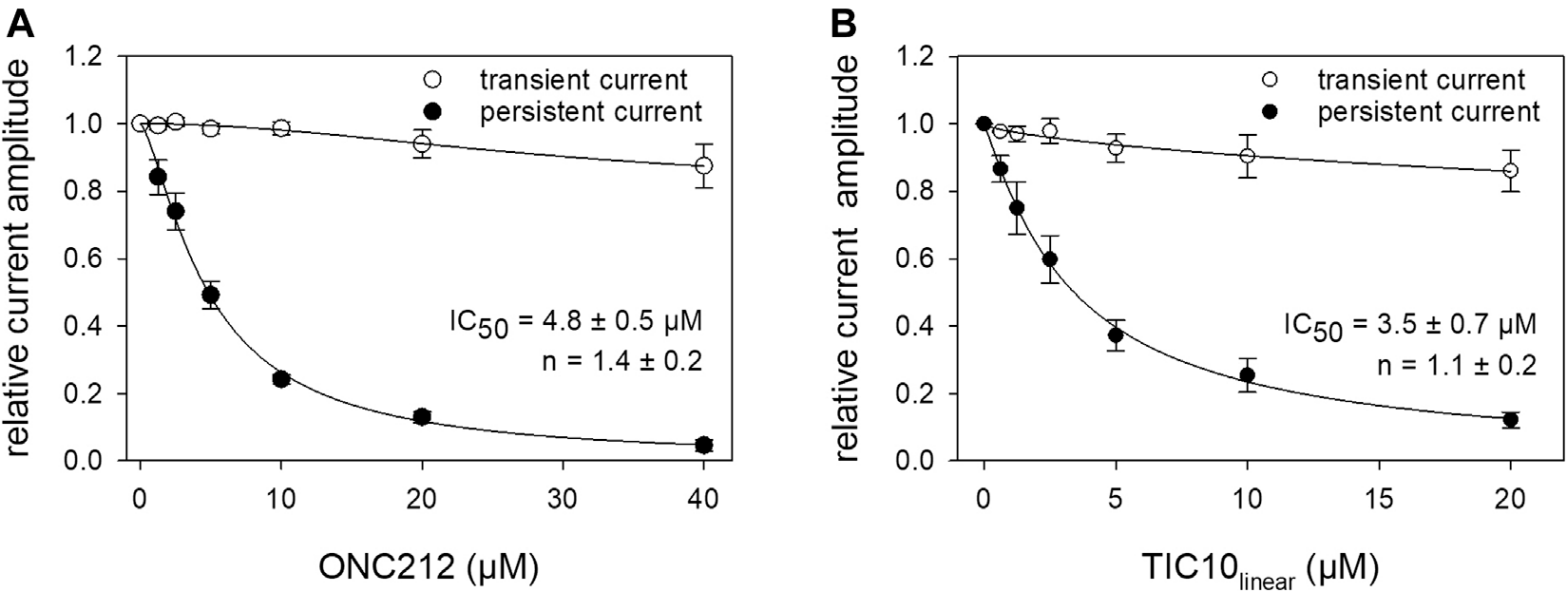
Interaction of imipridone derivatives with the open state. The interaction with the open state was analyzed by means of the WCW mutant. Experimental scheme as illustrated by Figure 12A. Relative transient and persistent current amplitudes are given in relation to the concentration of ONC212 **(A)** or TIC10_linear_ **(B)**.

Beside TIC10 angular, two other imipridones were of special interest. These were the linear isoform of TIC10 (TIC10_linear_) and a fluorinated derivative, called ONC212 (Figure 1). Concerning the anti-tumoral activity, TIC10_linear_ is nearly inactive, while ONC212 is more than 10 times more effective than TIC10 angular (Lev et al., 2017; Wagner et al., 2017; Nii et al., 2019). With respect to our investigations, we were interested to investigate if this anti-tumoral behavior is paralleled in the interaction with the hNa_v_1.5. For this purpose, only selected protocols were used. The experimental data show that in case of ONC212 the affinity for the slow inactivated state (according to Figure 8B) is nearly identical to that of TIC10 angular while TIC10_linear_ was about five-times less effective (Figure 14). In particular, the affinity for ONC212 was 2.5 ± 0.7 µM (Hill-coefficient 1.5 ± 0.1) and that for TIC10_linear_ was 11.2 ± 1.3 µM (Hill-coefficient 1.0 ± 0.1). In contrast to TIC10_angular_, the time course for block development had to be described for both compounds with two time constants (Figure 15). Data, listing the time constants and the relative amounts of the individual terms are summarized by Table 2. Due to the additional fast component, it was obvious that the derivatives might additionally affect fast inactivation. Indeed, this was the case for both compounds with a stronger effect obtained for ONC212 (Figure 16). ONC212 (10 µM) shifted mid points of fast inactivation by −4.7 ± 0.4 mV and TIC10_linear_ (10 µM) by −3.0 ± 0.4 mV. The possible interaction with the open state was analyzed using the WCW mutant. Both derivatives revealed here affinities close to TIC10_angular_ (Figure 17). In particular, for 10 µM ONC212, the calculated value was 4.8 ± 0.5 µM (Hill-coefficient: 1.4 ± 0.2); the corresponding value for 10 µM TIC10_linear_ was 3.5 ± 0.7 µM (Hill-coefficient 1.1 ± 0.2). Altogether, it turned out, that the anti-tumor potency of the analyzed compounds was only partly mirrored in individual experimental designs used to analyze their interaction with the hNa_v_1.5.

**TABLE 2:** Time-dependent interaction of imipridone derivatives with the inactivated state of the hNa_v_1.5

	ONC212	Amount	TIC10 linear	Amount
2.5 µM				
τ_1_ (s)	0.2 ± 0.06	8.8 ± 4.5%	0.2 ± 0.02	10.6 ± 3.1%
τ_2_ (s)	4.6 ± 0.95	57.4± 6.6%	6.7 ± 1.24	21.6 ± 4.5%
10 µM				
τ_1_ (s)	0.1 ± 0.03	45.2 ± 6.5%	0.1 ± 0.02	29.7 ± 9.7%
τ_2_ (s)	2.2 ± 0.27	46.9 ± 7.2%	5.0 ± 1.14	37.4 ± 4.9%

## Discussion

TIC10, later renamed to ONC201, belongs to a novel class of anti-cancer compounds called imipridones (Allen et al., 2016; Anderson and Scott, 2017). Among this group, several derivatives exist with a varying efficacy concerning anti-tumor activity (Wagner et al., 2017). There are several mechanisms of action that were reported to contribute to the anti-tumor activity of TIC10. First, the induction of TRAIL (Allen et al., 2013). Second, an interference with the dopamine receptor D_2_ (Kline et al., 2018). Third, inhibition of oxidative phosphorylation by hyperactivation of the mitochondrial caseinolytic protease P (ClpP) (Ishizawa et al., 2019). Here, we described an as far unknown pharmacological property of TIC10, which might contribute to its anti-tumor activity: TIC10 is a potent blocker of VGSCs. We determined that it interacts with the hNa_v_1.5 in a state- but not use-dependent manner. The interaction of TIC10 with different channel states occurs well within the concentration range in which it is used in tumor therapy.

### Voltage-Gated Sodium Channels and Cancer

The functional expression of VGSCs in tumor cells has initially been described for prostatic cancer cells (Grimes et al., 1995). Meanwhile, corresponding observations have been made for many other cancer cells. Altogether, there is no doubt that voltage-gated sodium channels play an important role in metastasis, especially in the processes controlling migration and invasion of tumor cells. Accordingly, blockade of VGSCs or reducing their expression level is expected to have beneficial effects in tumor therapy (Djamgoz and Onkal, 2013; Djamgoz et al., 2019).

In the past, the occurrence of VGSCs has been associated with excitable cells where they are required for generating action potentials. Meanwhile, mRNA encoding for this type of channel has been detected in a broad variety of non-excitable cells, including cancer cells. In the latter, mRNA levels of VGSCs can be upregulated more than 1000-fold, with the amount of VGSCs directly correlating to their metastatic potency (Fraser et al., 2005). Of note, the functional expression of VGSCs in tumor cells is much less than mRNA, but even strong enough to make these cells potentially excitable. However, excitability does not seem to be of relevance VGSCs play in tumorigenesis (Djamgoz et al., 2019). Moreover, it is their capability to be selectively permeable for sodium ions, whereby an increased level of the concentration of intracellular sodium ions results. Indeed, an elevated concentration of sodium ions in the tissue and in the cytosol of malignant cells has been found (Ouwerkerk et al., 2007; Li et al., 2015). It is evident that increased intracellular sodium levels cannot be achieved by the minute amount of sodium influx, which would result from brief channel openings (Roger et al., 2004; Carter and Bean, 2009). Aside from this, such activations are very unlikely to happen regarding the rather positive resting membrane potential (range: −40 to −20 mV) of these cells, which makes the VGSCs not available for activation (Roger et al., 2015). Once more, the relative positivity of the resting membrane potential of the analyzed cell lines correlates with their metastatic potency (Fraser et al., 2005). Thus, two ways for a permanent sodium influx are in focus: first, a window current in the range from −60 to −20 mV resulting from an incomplete overlap of the VGSCs activation and inactivation curves, with the maximum current at the more negative end of the resting potential range observed in tumor cells (Djamgoz and Onkal, 2013). Second, a persistent, non-inactivating sodium current (INaP) the activity of which is not restricted to a narrow potential range as it is also observed at depolarized membrane potentials (Maltsev et al., 1998). This latter current is also enhanced under hypoxia, a condition commonly observed in the tumor environment (Ju et al., 1996; Brahimi-Horn and Pouysségur, 2007). All in all, this persistent sodium current is favored as the base for the increased intracellular concentration of sodium ions which in turn activates the sodium-hydrogen exchanger 1 (NHE1). By the export of protons, an extracellular acidification is achieved, which is a prerequisite for proteolytic enzymatic activity providing the basis for invasiveness (Djamgoz et al., 2019). A pharmacological hallmark of the INaP is, that it can be blocked by almost all blockers of VGSCs as this current flows through the same channels as the transient, fast inactivating current (INaT) (Urbani and Belluzzi, 2000). Furthermore, there are also drugs (mexiletine, flecainide, ranolazine) which preferentially interact with this state while being less effective in other settings (Wang et al., 2003, 2004, 2008). However, a mere selectively targeting of the INaP is not a sufficient criterion for choosing and applying a sodium channel blocker as the different interactions of a particular blocker must be evaluated in toto.

### Impact of TIC10_angular_

TIC10 blocked the hNa_v_1.5 in a state-dependent manner. The interaction with the resting state was very low (around 600 µM), while the affinity to the inactivated state was about 2 µM. The ratio in affinity for the resting versus the inactivated state is generally regarded as a safety margin, the higher the better (Ilyin et al., 2005). To be judged as safe, the ratio should be at least 50-fold. In case of TIC10, the ratio is about 300-fold, which rates TIC10 as a safe drug in this setting. Next, the interaction of TIC10 with the hNa_v_1.5 happens in a slow manner whereby an interaction with INaT is rather unlikely. This applies to a single activation as well as for repeated activations as observed in the test analyzing for a use-dependent behavior. Concerning heart activity, this is a favorable property, as affecting INaT would potentially be proarrhythmic due to an intracardial conduction delay. Indeed, a prolongation of the QRS complex can be observed by current reductions as little as 10% (Poulin et al., 2014). In this way, it has been proposed earlier that an ideal VGSC blocker should only block the persistent current (INaP) while leaving the transient current (INaT) unaffected (Noble, 2006). The main problem for the analysis of drug effects on the INaP consists of its small size, which in wild-type channels is almost less than 1% compared to the corresponding INaT. Therefore, different experimental approaches have been developed in order to amplify its size. These include enzymatic or chemical treatment, the application of toxins, or the use of inactivation-deficient mutants (Grant et al., 2000; Belardinelli et al., 2013; Wang et al., 2013). However, it should be kept in mind, that all these manipulations might also have unspecific effects on drug interaction (Föhr et al., 2021). We performed our experiments with an inactivation-deficient mutant. Again, INaT was hardly affected while INaP revealed an affinity of about 4 μM, which is well beyond the maximal concentration found in the plasma of treated patients (Allen et al., 2016). In further experiments, we obtained hints that the interaction of TIC10 with the hNa_v_1.5 most probably happens via the binding site for local anesthetics as the affinity of the corresponding mutant was more than 20-fold lower as for wild-type channels. Thus, TIC10 uses the same mechanism/interaction site as the majority of blockers of VGSCs.

These findings make it unlikely that TIC10 can also discriminate between the adult and neonatal form of the hNa_v_1.5. Of course, a drug which blocks the neonatal form while leaving the adult form unaffected would be highly desired (Roger et al., 2004; Fraser et al., 2005). An important point here is that tumor cells, which express hNa_v_1.5 channels, preferentially express the neonatal form, whereas the neonatal form is expected to be absent to a large extent from normal adult tissue (Onkal and Djamgoz, 2009; Yamaci et al., 2017). So far, to the best of our knowledge, excepting a polyclonal antibody, no drug is known to discriminate between the adult and neonatal form of the hNa_v_1.5, including our previous study about the inhibitory effect of IC261 on these channels (Föhr et al., 2017). The main point here is that the two forms differ by only seven amino acids in segment three and four of domain I introducing one additional positive charge whereby amongst others channel activation will be affected (Walzik et al., 2011). However, as blockers of VGSCs, which affect channel activation, are extremely rare, it remains challenging to discover or design corresponding drugs.

Furthermore, it is tentative to speculate that the inhibitory action of TIC10 might not be restricted to the hNa_v_1.5 as state-dependent blocker often reveal a low isotype specificity (Ilyin et al., 2005). This might be of importance as almost all isoforms have been reported to occur in different tumors, whereby the isoform itself seems not to be critical for the biological effect in cancer (Roger et al., 2015). Furthermore, all VGSCs expressed in cancer cells also reveal an INaP irrespective of the subtype, at least under hypoxic conditions (Djamgoz et al., 2019).

### Impact of Imipridone Derivatives

Thus far, our results indicate that the concentration used for anti-tumor therapy and effective sodium channel-blocking activity of TIC10 (throughout used for TIC10_angular_) happen in a similar concentration range (a weekly oral dose of 625 mg TIC10 causes a maximal plasma concentration of 4.3 µM (Allen et al., 2016)). To investigate whether there might be a correlation between these two properties, we tested two other derivatives, which were either inactive or more potent than TIC10 concerning their anti-tumor activity. These were TIC10_linear_, an isomer of TIC10, which is completely inactive and ONC212, a fluorinated derivative which is at least 10 times more potent than TIC10 (Lev et al., 2017; Wagner et al., 2017; Nii et al., 2019). Our data clearly demonstrated that ONC212 has about the same potency as TIC10 angular while TIC10_linear_ was less effective, especially in the protocol analyzing for slow inactivation. This poses the question, which property of the sodium channel is most important for its ability to support tumor cells. So far, the persistent sodium current is regarded as most important (Djamgoz et al., 2019). In this case all imipridone derivatives are expected to be equally effective as they suppress the “persistent current” with almost identical efficacy. However, this is not reflected by the anti-tumor activity of the different imipridone derivatives. Clearly, it must be taken into account that the anti-tumor activity refers to the ability of a drug to induce TRAIL, leading to cell death or affecting cell proliferation, whereas the blockade of sodium channels targets migration and invasion of tumor cells but not proliferation (Wagner et al., 2017). In this context, it is noteworthy that sodium channel blockers cannot completely suppress invasion. If sodium channels are totally inhibited, invasiveness is reduced only by about 35%. Another point is that probably other channels/transporters contribute to an increase in intracellular sodium concentration (Leslie et al., 2019). Evidently, in these cases, invasiveness cannot be suppressed by blockers of VGSCs (Driffort et al., 2014; Roger et al., 2015). This also applies to tumor cells, which do not express VGSCs. Finally, even though imipridones affect different aspects of tumor progression, they do not eradicate tumors (Wang and Dougan, 2019).

### VGSCs Blocker and Cancer

The discovery of an unexpected anti-tumor activity of a drug often starts with the observation of an obviously diminished incidence for cancer under a selected medication. A prominent example here is metformin, commonly used for treating diabetes type 2, for which the anti-tumor properties have been confirmed by many retrospective analysis (Evans et al., 2005; Libby et al., 2009). Similarly, the outcome from the pharmacological treatment of neurological disorders has been analyzed. From most investigations, a positive correlation with respect to a beneficial outcome is reported (Reddy et al., 2015; Zhuo et al., 2019). However, there are also inverse associations (Takada et al., 2016). As many of the above-mentioned drugs operate as blockers for VGSCs, well-known blockers of this class are under investigation in different clinical studies for purposes of drug repurposing (Leslie et al., 2019). The list includes lidocaine, valproate, bupivacaine, or ranolazine. Interestingly, some of them preferentially target the persistent current (INaP) (Taverna et al., 1998; Wang et al., 2008). Beside this, the search for new blockers of VGSCs with respect to its potential application as a supplement in the tumor therapy is ongoing (Wang and Dougan, 2019). However, it should be kept in mind that beyond VGSCs many other ion channels and transporters might be of importance as supplements in tumor therapy (Cuddapah and Sontheimer, 2011; Biasiotta et al., 2016; Prevarskaya et al., 2018).

## Conclusion

TIC10 is a potent blocker of hNa_v_1.5, an important VGSC. Half-maximal effects occur well within the concentration range used for tumor therapy. Thus, blockade of VGSCs might contribute to the anti-tumor activity of TIC10.

## Data Availability

The raw data supporting the conclusions of this article will be made available by the authors, without undue reservation.
